# An intelligent platform for ultrasound diagnosis of thyroid nodules

**DOI:** 10.1038/s41598-020-70159-y

**Published:** 2020-08-06

**Authors:** Heng Ye, Jing Hang, Xiaowei Chen, Di Xu, Jie Chen, Xinhua Ye, Dong Zhang

**Affiliations:** 1 The MOE Key Laboratory of Modern Acoustics, Department of Physics, Nanjing University, Nanjing, 210093 China; 2 Department of Ultrasound, The First Affiliated Hospital of Nanjing Medical University, Nanjing, 210029 China; 3 Department of Geriatrics, The First Affiliated Hospital of Nanjing Medical University, Nanjing, 210029 China; 4 The State Key Laboratory of Acoustics, Chinese Academy of Science, Beijing, 10080 China

**Keywords:** Biomedical engineering, Cancer

## Abstract

This paper proposed a non-segmentation radiological method for classification of benign and malignant thyroid tumors using B mode ultrasound data. This method aimed to combine the advantages of morphological information provided by ultrasound and convolutional neural networks in automatic feature extraction and accurate classification. Compared with the traditional feature extraction method, this method directly extracted features from the data set without the need for segmentation and manual operations. 861 benign nodule images and 740 malignant nodule images were collected for training data. A deep convolution neural network VGG-16 was constructed to analyze test data including 100 malignant nodule images and 109 benign nodule images. A nine fold cross validation was performed for training and testing of the classifier. The results showed that the method had an accuracy of 86.12%, a sensitivity of 87%, and a specificity of 85.32%. This computer-aided method demonstrated comparable diagnostic performance with the result reported by an experienced radiologist based on American college of radiology thyroid imaging reporting and data system (ACR TI-RADS) (accuracy: 87.56%, sensitivity: 92%, and specificity: 83.49%). The automation advantage of this method suggested application potential in computer-aided diagnosis of thyroid cancer.

## Introduction

Thyroid cancer is the most common endocrine cancer, and its incidence has increased rapidly worldwide, especially in Asian countries^1,2^. Most of thyroid cancers show as thyroid nodules, which are usually detected by chance in the neck examination with ultrasonography because of other disorders^3,4^. When using high-resolution ultrasound, the prevalence of thyroid nodules is as high as 19–68% in a randomly selected population. Since most of the nodules are benign and the percentage of malignant ones is relatively low (7–15%), it is of great importance to distinguish benign and malignant thyroid nodules^1,5,6^. When doctors notice the presence of nodules, they will do a systematical assessment of the thyroid gland. It includes a set of bioanalysis of thyroid from blood tests, such as thyroxine (T4), triiodothyronine (T3), etc. But this usually cannot predict whether it is a benign or malignant nodule^7,8^. With the development of high-frequency ultrasound technology, systematic ultrasound examination of the neck can be carried out to identify the nature of the nodules^9^. This examination allows doctors to measure the number, size, and shape of nodules and to detect other possible abnormalities. Thyroid ultrasound provides information about the structure and characteristics of the nodules, which is helpful in the diagnosis of various types of thyroid nodules, including composition, echo, shape, margin and echoic foci^10,11^. Yet, this technique is also based on subjective assessments, ultrasound-guided fine needle aspiration (FNA) is recommended for the differential diagnosis of thyroid benign and malignant nodules^1^.

Deep convolution neural network (DCNN)^12–14^ is a kind of artificial intelligence method, which has been applied in more and more research fields^15–17^. It has new applications in dermatology^18^, ophthalmology^19^, radiology^20,21^ and other fields^22–24^. In recent years, the research of DCNN in the field of radiology shows that the performance of this algorithm is equivalent to that of radiologist. With the continuous development of this field, the possible types and quantities of deep learning are also increasing^25^. Compared with the traditional feature extraction method, DCNN method directly extracts features from the data set without the need for segmentation and complex manual operations^26,27^.

The objective of the current work was to design a computer-aided system based on DCNN to automatically classify the benign and malignant nodules based on thyroid ultrasound images. With B mode ultrasound data, this method aimed to combine the advantages of morphological information provided by ultrasound and convolutional neural networks in automatic feature extraction and accurate classification. The validity of this method was verified by comparing the current experimental results with results obtained by an experienced radiologist based on American college of radiology thyroid imaging reporting and data system (ACR TI-RADS)^28–30^.

## Results

A total of 1,810 images from 1,452 subjects were obtained, of which 840 were malignant and 970 were benign. Detailed information of the collected cases are shown in Table 1. The total number of malignant nodule images is 840 (1,810, 46.4%), which includes 740 (1601, 46.22%) malignant nodule images in training group and 100 (209, 47.8%) malignant nodule images in testing group. The percentage of malignant and benign nodules between the training group and the test group is not statistically significant (Table 2).

**Table 1:** Detailed information of collected cases.

Results	Diagnostic method	Detailed results	Number
Benign	FNA	Bethesda II	132
	US findings	Cystic, spongy	713
	Surgery	Nodular goiter	13
		Adenoma	106
		Hashimoto’s thyroiditis	3
		Focal thyroiditis	3
Malignant	FNA	Bethesda VI	293
	CNB	Squamous cell carcinoma	1
		Anaplastic thyroid carcinoma	1
		Primary thyroid lymphoma	4
	Surgery	Papillary carcinoma	525
		Medullar carcinoma	4
		Follicular adenocarcinoma	12

*FNA* fine needle aspiration, *CNB* core-needle biopsy.

**Table 2:** The basic information of collected cases.

Nodules	Training set	Test set
Benign	861 images Female 494, 47.2 ± 13.4 years old Male 126, 50.8 ± 15.8 years old	109 images Female 61, 46.7 ± 12.2 years old Male 15, 49.5 ± 14.6 years old
Malignant	740 images Female 441, 42.7 ± 11.2 years old Male 182, 41.7 ± 11.9 years old	100 images Female 59, 41.8 ± 12.2 years old Male 31, 43.7 ± 10.4 years old

Mean data are mean ± standard deviation.

**Figure 1:**
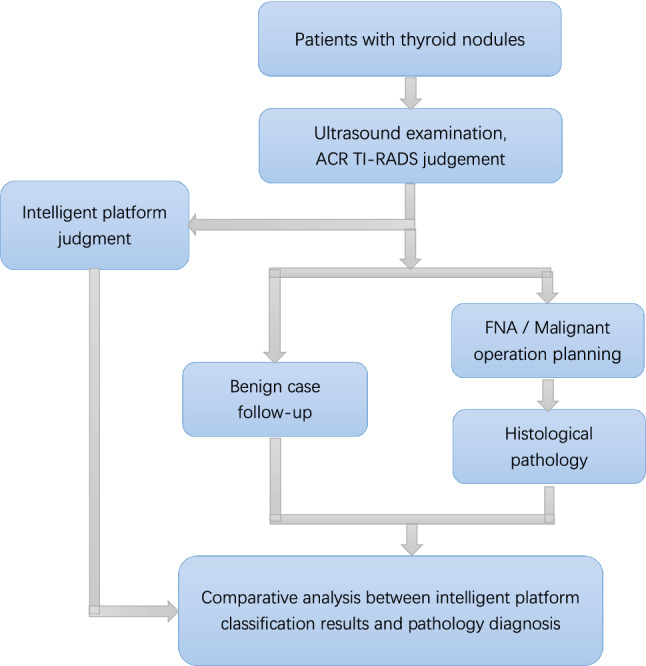
Inclusion criteria for the initial cohorts and experiment procedure for the final study cohorts.

The experimental steps are illustrated in Fig. 1. In the current work, the proposed DCNN model was used to analyze the thyroid ultrasound images. FNA and surgical results were taken as the reference.

For causing no additional workload for the radiologist, we used a bound box of a nodule by enclosing calipers (used in clinics for nodule measurement), and we did not need to draw the boundary of the nodule by a radiologist. The deep convolution neural network VGG-16^31^ for large-scale target recognition was evaluated, and the nodule recognition based on ultrasound image was fine tuned.

We performed validation of the performance of the classifier using a nine folded cross validation. As shown in Table 3, for the training set of differentiating benign and malignant nodules, the areas under the receiver operating characteristic curves (AUC) of the algorithm is 0.9054 (95% confidence interval (CI) 0.8773, 0.9336). The accuracy is 86.27% (95% CI 84.11%, 88.43%), the sensitivity was 87%, and the specificity is 86.42% (95% CI 83.10% 89.74%).

**Table 3:** The result of training set.

Round	Accuracy (%)	Sensitivity (%)	Specificity (%)	AUC
1	90.34	89.77	90.91	0.9412
2	82.74	78.21	86.67	0.8615
3	84.15	81.08	86.67	0.877
4	89.44	85	93	0.9159
5	85.56	94.05	78.12	0.9149
6	87.63	90.20	84.78	0.9297
7	84.92	82.24	88.04	0.9051
8	88.54	90.29	86.52	0.9561
9	83.11	83.33	83.06	0.8474

**Figure 2:**
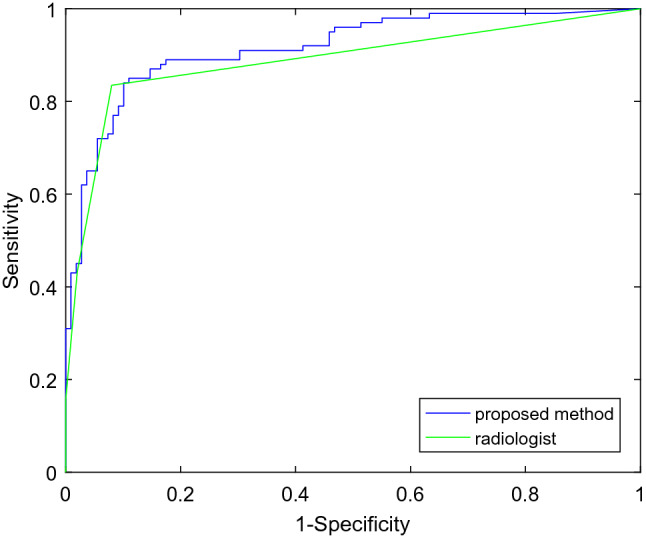
AUC of the proposed algorithm on the test dataset.

As shown in Fig. 2, for the test set of differentiating benign and malignant nodules, the AUC of our proposed method is 0.9157, the accuracy is 86.12%, the sensitivity is 87%, and the specificity is 85.32%. The AUC of the experienced radiologist diagnosis is 0.8879. The cut off value of TI-RADS is 4 reported by the radiologist, which is corresponding to the top-left point on receiver operating characteristic curve. The accuracy, sensitivity and specificity reported by the radiologist according to ACR TI-RADS are 87.56%, 92% and 83.49% respectively. Statistical analysis^32,33^ shows that there is no significant difference between our algorithm and the result reported by the experienced radiologist (p > 0.1).

## Discussion

Ultrasound diagnosis of thyroid nodules is time-consuming and labor-intensive, and has interreader variability. In this research, we developed a deep learning algorithm to provide management recommendations for thyroid nodules, based on ultrasound image observations, and compared the results with those obtained by radiologist who follows the guidelines of ACR TI-RADS. With the thyroid nodule classification system proposed in this paper based on deep neural networks, experimental results on ultrasound images indicated that this method would achieve comparable classification performance to the result reported by the experienced radiologist. In the present work, we applied deep neural network to the dataset (1601/209 for training/testing). 1601 training data included 861 benign nodule images and 740 malignant nodule images. The 209 data included 109 benign nodule images and 100 malignant nodule images. The experimental results showed that the accuracy, the sensitivity and the specificity of this method achieved 86.12%, 87% and 85.32%, respectively.

Our findings supported increasing evidence that deep learning could be applied to the thyroid clinical diagnosis. After training, through a similarity activation map analysis, the DCNN model could be used to pinpoint malignant thyroid nodules. DCNN models, together with machine learning methods based on traditional feature extraction were used to identify the malignancy of thyroid nodules with ultrasound images. Ma et al.^24^ used DCNN to analyze 8,148 hand-labeled thyroid nodules and obtained 83.0% (95% CI 82.3–83.7) thyroid nodule diagnostic accuracy. This experiment required big data set for training. Xia et al.^34^ obtained the accuracy of distinguishing benign and malignant nodules by 87.7%, with extreme learning machine and radiology features collected from 203 ultrasound images of 187 thyroid patients. This method had a relatively lower specificity and need to draw nodule boundary by radiologist, which brought a lot of work to doctors. Pereira et al. reported that the accuracy of the DCNN model in distinguishing 946 malignant and benign thyroid nodules from 165 patients was 83%^35^. Chi et al.^23^ used the imaging features extracted by deep convolutional neural network and performed binary tasks for classifying TI-RADS category 1 and category 2 from the other categories, and reached more than 99% accuracy. Although the performance seemed to be excellent, it was a greatly simplified task of predicting category 1 and category 2. Also, the research subjects did not have FNA and surgery results to be compared with.

In the present study, all the patients with thyroid cancers in training and test data sets had FNA or surgery results. Furthermore, to avoid additional workload for the radiologist, we did not need to draw the boundary of the nodule by a radiologist. In another study of Mateusz Buda et al. 2019, 1,377 thyroid nodules were used in 1,230 patients with complete imaging data and clear cytological or histological diagnosis^36^. For 99 test nodules, the proposed deep learning algorithm achieved a sensitivity of 13/15 (87%: 95% confidence interval: 67%, 100%), which was the same as the expert and higher than 5 of 9 radiologists. The specificity of the deep learning algorithm achieved 44/84 (52%; 95% CI 42%, 62%)), which was similar to the consensus of experts (43/84; 51%; 95% CI 41%, 62%; p = 0.91), higher than the other 9 radiologists. The average sensitivity and specificity of the 9 radiologists were 83% (95% CI 64%, 98%) and 48% (95% CI 37%, 59%). Our experiment had a comparative sensitivity and higher specificity.

In summary, the proposed DCNN diagnosis algorithm could be used to effectively classify benign and malignant thyroid nodules, and exhibited comparable diagnostic performance to the results reported by the experienced radiologist according to TI-RADS. This method might enable potential applications in computer-aided diagnosis of thyroid cancer. However, the present study still had some limitations. For instance, we did not find the accuracy of the computer-aided platform proposed in the work had connection with tumor size and cancer subtypes. The number of cases enrolled in the current study were small. More types of patients should be validated, and the accuracy of the proposed model should be further verified and improved.

## Methods

### Research cohort

This retrospective study was approved by the institutional review board of the First Affiliated Hospital of Nanjing Medical University and informed consent was obtained from all patients. All study methodologies were carried out in accordance with relevant guidelines and regulations. From January 2018 to September 2019, a group of patients with thyroid nodules who took ultrasound examination before surgery or biopsy were included in the retrospective study. The inclusion criteria were determined as follows: (a) age > 18 years; (b) not received hormone therapy, chemotherapy, or radiation therapy; (c) thyroid nodule diameter > 5 mm. Images without diagnostic, indeterminate cytologic or histological results were excluded. The diagnosis of a malignant nodule was made when malignancy was confirmed on surgical specimen by core-needle biopsy (CNB) or FNA cytology. A benign nodule was made when any one of the following criteria was met: (a) confirmation using a surgical specimen; (b) benign FNA cytology findings; or (c) US findings of very low suspicion^9^; and (d) Cystic or almost complete cystic nodules and spongy nodules (mainly composed of more than 50% of the small cystic space).

The database of 1,810 thyroid disease images was evaluated by two experts. B-ultrasonic examination was carried out by several commercial US equipment: (1) Esaote MyLab twice (Genova, Italy). (2) GE LOGIQ E9 (USA). (3) Philips EPIQ 5 (Amsterdam, the Netherlands). (4) Philips EPIQ 7 (Amsterdam, Netherlands) (5) Siemens S3000 (Buffalo, USA). (6) Supersonic imagine (aixplorer, Aix-en-Provence, France), etc. A 5–13 MHz wide-band linear array probe was utilized with a central working frequency of 7.5 MHz. The patient was placed in a supine position, expose the anterior cervical region, and then scanned laterally, longitudinally, and obliquely.

### Pathological reference

**Figure 3:**
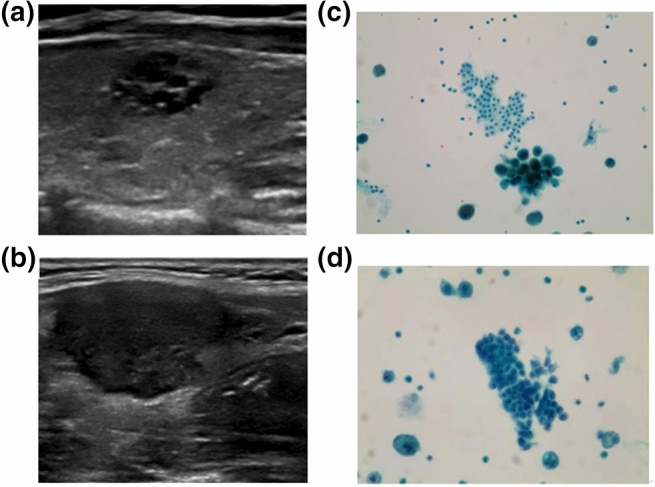
The B mode ultrasonography and FNAs of thyroid nodules: (**a**) B mode ultrasound image of benign thyroid nodule; (**b**) B mode ultrasound image of malignant thyroid nodule; (**c**) micrograph of a FNA smear of benign thyroid nodule with magnification power 200; and (**d**) micrograph of a FNA smear of malignant thyroid nodule with magnification power 400.

It is known that ultrasound-guided FNA has high specificity and sensitivity in the diagnosis of thyroid benign and malignant nodules, so that it can be used as a reference for the differential diagnosis of thyroid benign and malignant nodules. Therefore, in the present work, FNA was taken as the reference after B-mode ultrasound diagnosis. The final pathological diagnosis of a benign or malignant thyroid nodule was classified if a thyroid nodule had a benign or malignant cytology (or histology, if available). Under the guidance of ultrasound of interventional radiologist, 25G needle was used. After the location of the nodule was determined under the guidance of ultrasound, several samples in the nodule were obtained by using the needle in the ultrasound scanning plane. Three or four biopsies were fixed in BD CytoRich Red Preservative fluid (Becton, Dickinson and company, Mebane, USA), and then sedimentation-based cytologic examination was taken. All slides were reviewed and explained by three experienced cytotechnologists who reported thyroid cytopathology with reference to the Bethesda system. If the nodule had undergone core needle biopsy or surgical resection, the histologic results should be used instead of cytological examination. Figure 3a shows the B mode ultrasound image of benign thyroid nodule. Figure 3b shows the B mode ultrasound image of malignant thyroid nodule. Figure 3c shows the FNA smear micrograph of benign nodule. Figure 3d shows the FNA smear micrograph of malignant nodule. Cytologic images were obtained based on the Pap staining.

### Algorithm

In this paper, deep convolution neural network VGG-16 was fine-tuned and evaluated based on ultrasound image for the thyroid nodule diagnosis. Convolutional neural network included five convolution and pooling operation modules for extracting complex features from each input image. These features were flattened into a single vector. The output of the model was a collection of continuous variables that represented the predicted probabilities for each category (range 0.0–1.0) and were treated as discrete probability distributions. The final classification was calculated as a probability-weighted classes.

**Figure 4:**
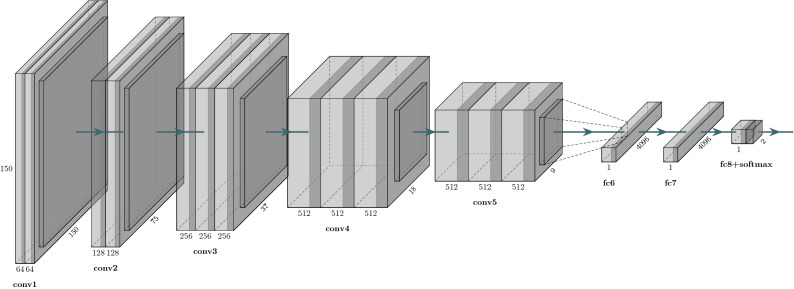
Network Structure of the intelligent platform.

The input of this network was subjected to a stack of convolutions and 3 × 3 filter was pushed to a depth of 16 to 19 weighted layers. A bunch of convolutions were followed by three fully connected layers (viz., 16 layers with learnable weights, 13 convolutions and 3 fully connected layers). These networks were fine-tuned using training sets containing benign and malignant samples to identify nodules. This was done by extracting all layers except the last fully connected layer from the pre-trained network and adding new fully connected layers and softmax. The obtained supervised training performed nodule classification tasks with expected results and reduced computational complexity. Figure 4 is the Network Structure of the intelligent platform.

### Statistical analyses

We performed a t-test of the hypothesis that the data in the vector X came from a distribution with mean zero, and returned the result of the test in H. H = 0 indicated that the null hypothesis could not be rejected at the 5% significance level. H = 1 indicated that the null hypothesis should be rejected at the 5% level. The data were assumed to come from a normal distribution with unknown variance.
